# E-Cigarette- or Vaping-Associated Lung Injury: An Unprecedented Enigma

**DOI:** 10.31486/toj.20.0015

**Published:** 2020

**Authors:** Abbas Ali Hussain, Rabia Sarwar, Amber Tahir

**Affiliations:** ^1^ Jinnah Sindh Medical University, Karachi, Pakistan; ^2^ Dow University of Health Sciences, Karachi, Pakistan

## TO THE EDITOR

The practice of smoking e-cigarettes—called vaping—has increased rapidly worldwide. The number of people who vape increased from approximately 7 million in 2011 to 41 million in 2018, with a corresponding threat to public health.^1^ The United States has experienced an outbreak of vaping-associated lung injury (VALI) with as many as 2,711 cases documented in the period of March 2019 through January 2020 and 60 confirmed deaths.^2^

According to a study published in *The Lancet*, VALI is associated with a variety of constitutional, respiratory, and gastrointestinal symptoms; the most common are fever, dyspnea, nausea, and emesis. VALI should be suspected in individuals who used e-cigarettes for at least 90 days before the onset of symptoms, whose plain chest X-ray or chest computed tomography scan shows pulmonary infiltrates, and who have no other known cause of infection.^3^ The development, progression, and severity of VALI depend on the duration of use and the amount of toxic substances inhaled. Many patients with VALI have admitted to using products containing nicotine and tetrahydrocannabinol (THC). Nicotine and THC are postulated as the main culprits for VALI, but no conclusions can be drawn because of the lack of scientific evidence. Further, e-cigarettes contain a variety of chemical products and flavors, so identifying a standardized causative agent has not yet been possible.^4^ According to the Centers for Disease Control and Prevention, people of all age groups have contracted VALI, although the majority appear to be teens and young adults.^2^

All of the currently reported VALI cases are in the United States, but the use of e-cigarettes is rising globally and begs for an immediate response from public health specialists across the world.^2^ Youth are the prime target of marketing efforts for products containing nicotine. Educating teenagers and young adults about this health issue is crucial.

In underdeveloped countries such as Pakistan, the prevalence of e-cigarette users has not yet been documented. According to a news report,^6^ young adults in Pakistan frequently use e-cigarettes; however, the government has not established an e-cigarette control policy, and e-cigarettes are sold without any restriction.^5,6^ Considering the hazards of vaping, authorities should propose and implement an awareness campaign encouraging abstinence and a ban of these products.^2,3,5^
